# Asynchronous Non-Fragile *H*_∞_ Control for Time-Delay Markovian Jump Singularly Perturbed Systems with Variable Quantization Density and DoS Attack

**DOI:** 10.3390/e28030317

**Published:** 2026-03-12

**Authors:** Yong Qin, Xiru Wu, Haolin Xiao, Lihong Huang, Yi Lu

**Affiliations:** 1 School of Artificial Intelligence and Manufacturing, Hechi University, Hechi 546300, China; yongqin2026@163.com; 2 School of Electronic Engineering and Automation, Guilin University of Electronic Technology, Guilin 541004, China; xiruwu@guet.edu.cn (X.W.); xiaohaolin@guet.edu.cn (H.X.); 3 Department of Mathematics and Computer Science, Changsha University, Changsha 410022, China; lhhuang@hnu.edu.cn

**Keywords:** Markovian jump singularly perturbed systems, time delay, non-fragile control, asynchronous, variable quantization density

## Abstract

This paper investigates the asynchronous non-fragile $H_{\infty }$ control problem for a class of Markovian jump singularly perturbed systems (MJSPSs) with time-varying delays. By applying a multi-layer structure method, a non-fragile controller with time delay is designed for the MJSPSs to adapt to disturbances caused by nonstationary quantization and DoS attacks. To model the asynchronous dynamics between the system and the controller mode, an independent Markov chain is employed to capture the asynchronous quantization and control behavior. By constructing mode-dependent Lyapunov–Krasovskii functions, sufficient conditions are derived to ensure stochastic finite-time exponential stability and $H_{\infty }$ performance under conditions of delay, singular disturbances, and quantization uncertainty. The effectiveness of the method is validated using an inverted pendulum system controlled by a DC motor, demonstrating its ability to achieve robust stability and performance in bandwidth-constrained network environments.

## 1. Introduction

In the field of control theory, Markov jump singularly perturbed systems (MJSPSs) are an important area of research because they can model complex dynamics in applications such as industrial automation, power systems, and unmanned vehicle networks. Delay phenomena are prevalent in networked control systems, industrial processes, and robotics, often leading to performance degradation, instability, and other undesirable behaviors, necessitating robust stability analysis and control strategy design [1,2]. MJSPSs are characterized by Markov chain-controlled random mode transitions and singular system structures with algebraic constraints, and the introduction of random dynamics and structural complexity further exacerbates these challenges [3]. These systems are widely applied to capture real-world phenomena such as equipment mode switching, power grid reconfiguration, and communication link changes.

The complexity escalates when MJSs incorporate singular system structures, also known as descriptor systems, which extend beyond standard state-space models by encompassing algebraic constraints. MJSPSs with time delays thus represent a class of complex systems that require advanced control strategies to ensure stability, regularity, and shock-free behavior. Among these strategies, $H_{\infty }$ control stands out as a robust method that excels at mitigating the worst effects of external disturbances and uncertainties on system performance [4]. However, traditional $H_{\infty }$ controllers typically assume precise implementation, and this assumption is often violated in practice due to controller parameter variations. Although control laws are implemented digitally, inaccuracies inevitably arise from finite word length (rounding errors) in processors, tolerances of analog components in A/D and D/A interfaces, and component aging. These factors lead to deviations between the designed ideal gains and the actual implemented gains. Wang G. et al. studied the guaranteed cost control of semi-MJSs in a network environment using event-triggered controllers based on the mode classifications [5]. This vulnerability highlights the need for non-fragile control design, which maintains robustness even in the presence of such defects [6].

Due to the inherent limitations of digital communication, various challenges arise, including quantization effects [7,8,9], network latency [10,11], and signal loss [12,13], which may lead to degraded system performance or even instability. Among these, quantization effects are particularly critical, as they enable efficient bandwidth utilization by mapping continuous signals to discrete values. Sector boundary techniques are commonly used to address quantization errors, treating them as bounded uncertainties within sectors [14]. Quantization control optimizes resource utilization by discretizing signals before controller input, making it indispensable in bandwidth-constrained environments. However, traditional quantization approaches often assume mode-independent or synchronous mode-dependent quantizers, as explored in [9,15,16]. These assumptions may not fully capture the dynamic nature of MJSs, where system modes switch randomly according to a Markov process. Asynchronous quantization, where the quantizer operates independently of system modes, has received limited attention, with notable exceptions in [17,18,19]. Specifically, Ref. [17] investigated asynchronous quantizers for MJSs, demonstrating their potential to enhance control flexibility. To address the limitations imposed by finite bandwidth in the cyber–physical systems, the raw measurement output data is quantized before entering the network [20]. Ref. [21] investigated the problem of asynchronous resilient $H_{\infty }$ dynamic state feedback control in a class of uncertain discrete-time switched nonlinear systems with input quantization. By using the time-delay asynchronous quantization control method, the stabilization problem of an uncertain discrete-time Markov jump power system subjected to deception attacks is studied [22]. Nevertheless, the integration of variable quantization density, which allows quantization levels to adapt dynamically to system requirements or communication conditions, remains underexplored in this context. Quantized measurement offers the promise of optimizing quantization precision, but its application to singular MJSs with time delays poses significant challenges due to the interplay of nonlinear dynamics, mode switching, and controller fragility.

Motivated by these challenges, this paper investigates asynchronous non-fragile $H_{\infty }$ control for time-delay MJSPSs with variable quantization density. The main contributions are outlined as follows:

1. A robust asynchronous non-fragile $H_{\infty }$ control framework is proposed to address variable quantization density in bandwidth-constrained networks, ensuring stability and performance under time delays, singular perturbations, and mode-switching uncertainties.
2. An asynchronous controller, governed by an independent Markov chain, is designed to achieve flexible mode-dependent control, while explicitly considering the impact of DoS attacks on system performance.
3. Sufficient conditions for the existence of the controller are derived, integrating quantized measurement to enhance robustness and stability against quantization errors, time delays, and singularly perturbed dynamics.

The paper is structured as follows: Section 2 formulates the discrete-time MJSPSs and provides preliminary definitions. Section 3 conducts stability and $H_{\infty }$ performance analysis. Section 4 validates the proposed approach through numerical experiments. Section 5 concludes the study with future research directions.

**Notation** **1.** *$R^{n}$ denotes an n-dimensional vector.* Pr *is the probability function. E denotes the mathematical expectation. |·| is the norm. I denotes the identity matrix.*

## 2. Problem Statement and Preliminaries

Consider the following time-delay MJSPSs consisting of *N* nodes:

(1) $$\left\{\begin{array}{c} x\left(k+1\right)=A_{\sigma (k)}E_{\epsilon }x\left(k\right)+B_{\sigma (k)}E_{\epsilon }\left(x\left(k\right)-\tau \left(k\right)\right)+C_{\sigma (k)}u\left(k\right)+D_{\sigma (k)}w\left(k\right),\\ y\left(k\right)=F_{\sigma (k)}x\left(k\right), \end{array}\right.$$

where $x\left(k\right)={\left[x_{1}^{T}\left(k\right),\;x_{2}^{T}\left(k\right)\right]}^{T}\in R^{n_{x}}$ is state vector with $n_{x}=n_{s}+n_{f}$; $x_{1}\left(k\right)\in R^{n_{s}}$ and $x_{2}\left(k\right)\in R^{n_{f}}$ denote the slow and fast state vectors. $u\left(k\right)\in R^{n_{u}}$ stand for the control input with the disturbance. $y\left(k\right)\in R^{n_{y}}$ is the measured output. $w\left(k\right)\in R^{n_{w}}$ is the external disturbance input. The external disturbance input $w\left(k\right)\in R^{n_{w}}$ is assumed to be an arbitrary signal in $w\left(k\right)\in l_{2}\left[0,\infty \right)$, which can represent stochastic environmental noises with finite energy. $A_{\sigma (k)},\;B_{\sigma (k)},\;C_{\sigma (k)},\;D_{\sigma (k)}$, and $F_{\sigma (k)}$ are known matrices. $E_{\epsilon }=diag\left\{I_{n_{s}},\;\epsilon I_{n_{f}}\right\}$ and ϵ is the SPP. The time-varying delay term τ(k) satisfies the constraint condition $0<\tau_{m}<\tau \left(k\right)\leq \tau_{M}$, where the upper and lower bounds of the delay $\tau_{m}$ and $\tau_{M}$ are both positive integers. The system mode is regulated by a discrete Markov chain (DMC): $\sigma \left(k\right)\in S_{1}=\left\{1,\;\ldots ,\;s_{1}\right\}$. The transition probability matrix (TPM) $\Pi =[\pi_{ab}]$ is given by

(2) $$\begin{array}{c} \mathrm{Pr}\left\{\sigma \left(k+1\right)=b|\sigma \left(k\right)=a\right\}=\pi_{ab},\;a,b\in S_{1}, \end{array}$$

where σ(k)≤0 and $\sum_{b=1}^{s_{1}}\pi_{ab}=1$.

**Figure 1:**
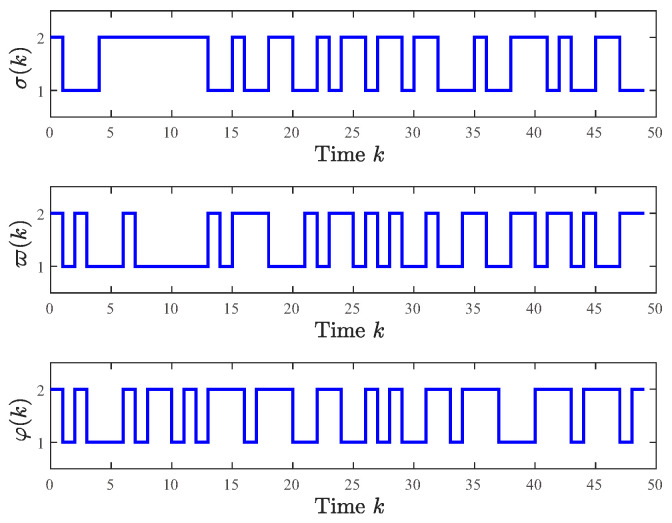
Markov jump mode of the system, quantizer, and controller.

In unreliable communication environments, the MJSPSs face problems such as data conflicts and communication congestion due to limited communication capacity. To address these challenges, this paper employs a quantized sensor communication protocol that determines which sensor can access the network at moment *k* through a scheduling mechanism. As illustrated in Figure 1, the measured output needs to be quantized before it is sent from the sensor to the controller via the shared communication network. The mode-dependent quantizer $q_{\varpi (k)}\left(\cdot \right)$ is formulated as follows:

(3) $$q_{s,\varpi (k)}\left(x_{s}\left(k\right)\right)=\left\{\begin{array}{cc} x_{s}^{l}, & \frac{x_{s}^{l}}{1+\sigma_{s,\varpi (k)}}<x_{s}<\frac{x_{s}^{l}}{1-\sigma_{s,\varpi (k)}},\\ 0, & x_{s}=0,\\ -q_{s}\left(-x_{s}\right), & x_{s}\leq 0 \end{array}\right.$$

where $\sigma_{s,\varpi (k)}=\frac{1-\rho_{s,\varpi (k)}}{1+\rho_{s,\varpi (k)}}$ with $\sigma_{s,\varpi (k)}\in \left(0,\;1\right)$. According to the sector bound uncertainty [7], one has

(4) $$q_{s,\varpi (k)}\left(x_{s}\left(k\right)\right)=\left(I+\Delta_{s,\varpi (k)}\right)x_{s}\left(k\right),$$

where $∥\Delta_{s,\varpi (k)}∥\leq \Lambda_{s,\varpi (k)}$. The quantizer mode is regulated by a DMC: $\varpi \left(k\right)\in S_{2}=\left\{1,\ldots ,s_{2}\right\}$. The TPM $\Phi =[\phi_{mn}]$ is given by

(5) $$\begin{array}{c} \mathrm{Pr}\left\{\varpi \left(k+1\right)=n|\varpi \left(k\right)=m\right\}=\phi_{mn},\;m,n\in S_{2}, \end{array}$$

where ϕ(k)≤0 and $\sum_{n=1}^{s_{2}}\phi_{mn}=1$.

Defining $\Delta_{\varpi (k)}=diag\left\{\Delta_{1,\varpi (k)},\;\Delta_{2,\varpi (k)},\ldots ,\Delta_{n_{x},\varpi \left(k\right)}\right\}$ and $\Lambda_{\varpi (k)}=diag\left\{\Lambda_{1,\varpi (k)},\Lambda_{2,\varpi (k)},\ldots ,\Lambda_{n_{x},\varpi \left(k\right)}\right\}$, it is obviously that Δ satisfy ${\left(\Delta_{\varpi (k)}\Lambda_{\varpi (k)}^{-1}\right)}^{T}\left(\Delta_{\varpi (k)}\Lambda_{\varpi (k)}^{-1}\right)\leq I$.

Based on the quantizer (3), the non-fragile feedback controller is designed as follows:

(6) $$\begin{array}{cc} u_{s}\left(k\right)= & \alpha \left(k\right)\left(K_{s,\varphi (k)}+\Delta K_{s,\varphi (k)}\right)E_{\epsilon }q_{s,\varpi (k)}\left(x\left(k\right)\right)\\ \; & +\left(K_{ds,\varphi (k)}+\Delta K_{ds,\varphi (k)}\right)E_{\epsilon }x_{s}\left(x\left(k\right)-\tau \left(k\right)\right) \end{array}$$

where $K_{\varphi (k)}$ is the controller gain. $\Delta K_{\varphi (k)}$ and $K_{ds,\varphi (k)}$ are the controller gain fluctuations that satisfy norm boundedness $\Delta K_{\varphi (k)}=U_{1\varphi (k)}O_{1}\left(k\right)V_{1\varphi (k)}$ and $\Delta K_{d\varphi (k)}=U_{2\varphi (k)}O_{2}\left(k\right)V_{2\varphi (k)}$. $U_{1\varphi (k)}$, $U_{2\varphi (k)}$, $V_{1\varphi (k)}$ and $V_{2\varphi (k)}$ are known time-varying Matrices, $O_{1}\left(k\right)$ and $O_{2}\left(k\right)$ are an unknown continuous function satisfying $O_{1}^{T}\left(k\right)O_{1}\left(k\right)\leq I$ and $O_{2}^{T}\left(k\right)O_{2}\left(k\right)\leq I$, used to characterize the uncertainty boundary of the attack. $\varphi \left(k\right)\in S_{2}=\left\{1,\ldots ,s_{3}\right\}$ can be governed by a DMC subject to TPM $\Psi^{\sigma (k+1)}=\left[\psi_{pq}^{\sigma (k+1)}\right]$ with $\psi_{pq}^{\sigma (k+1)}$ defined by

(7) $$\begin{array}{c} \mathrm{Pr}\left\{\varphi \left(k+1\right)=q|\varphi \left(k\right)=p\right\}=\psi_{pq}^{\sigma (k+1)},\;p,q\in S_{3}, \end{array}$$

where ψ(k)≥0 and $\sum_{q=1}^{s_{3}}\psi_{pq}^{\sigma (k+1)}=1$.

**Remark** **1.** *The random sequence α(k) obeys a Bernoulli distribution. It follows that Pr{α(k)=1}=α,Pr{α(k)=0}=1−α with α∈[0,1] being the frequency of attacks: (1) α(k)=1 implies that the transmission process is subjected to deception attacks, resulting in the removal of the desired measurement signal and its replacement with a bounded signal. (2) α(k)=0 indicates that the desired quantized signal can be successfully transmitted through the system.*

Define $q_{\varpi (k)}={\left[q_{1,\varpi (k)},q_{2,\varpi (k)},\ldots ,q_{n_{x},\varpi \left(k\right)}\right]}^{T}$ and $u\left(k\right)=[u_{1}\left(k\right),\;u_{2}\left(k\right),\ldots ,u_{n_{u}}\left(k\right)]$, and then

(8) $$\begin{array}{c} u\left(k\right)=\alpha \left(k\right)\left(K_{\varphi (k)}+\Delta K_{\varphi (k)}\right)\left(I+\Delta_{\varpi (k)}\right)E_{\epsilon }x\left(k\right)+\left(K_{d,\varphi (k)}+\Delta K_{d,\varphi (k)}\right)E_{\epsilon }x\left(x\left(k\right)-\tau \left(k\right)\right) \end{array}$$

**Remark** **2.** *The non-fragile controller u(k) incorporates both the state x(k) and a time-varying delay term x(k−d(k)). The controller gain consists of two components: The terms $\Delta K_{\varphi (k)}$ and $\Delta K_{d,\varphi (k)}$ represent the bounded fluctuations of the controller gains. These perturbations account for numerical rounding errors in digital implementation and physical parameter drifts in actuator circuits. Incorporating these uncertainties into the design ensures that the system maintains $H_{\infty }$ performance even when the controller is not implemented with infinite precision.*

**Remark** **3.** *It is noteworthy that the variable quantization density in this paper is regulated by a Markov chain. This modeling strategy aims to capture the stochastic nature of network bandwidth availability. In practical engineering, this corresponds to scenarios where senior network administrators allocate different quantization codebooks based on current traffic load. Although this is not a deterministic threshold-triggered adjustment, the Markov model provides a robust framework for stability analysis under random communication constraints.*

Let σ(k)=a, ϖ(k)=m, φ(k)=p. By substituting the controller (8) into the system (1), we obtain

(9) $$\begin{array}{cc} x(k+1) & =A_{a}E_{\epsilon }x\left(k\right)+\left(B_{a}+\left(K_{dp}+\Delta K_{dp}\right)\right)E_{\epsilon }\left(x\left(k\right)-\tau \left(k\right)\right)+\left(1-\alpha \right)C_{a}\left(K_{p}+\Delta K_{p}\right)\\ \; & E_{\epsilon }\left(1+\Delta_{m}\right)x\left(k\right)-\left(\alpha \left(k\right)-\alpha \right)C_{a}\left(K_{p}+\Delta K_{p}\right)E_{\epsilon }\left(I+\Delta_{m}\right)x\left(k\right)+D_{a}w\left(k\right) \end{array}$$

**Lemma** **1** **([9]).** *For given proper dimensioned matrices $W_{a}\left(a=1,2,3\right)$ that satisfies $W_{1}^{T}=W_{1}$, the following inequality holds:*

$$\begin{array}{c} W_{1}+W_{2}W_{3}+W_{3}^{T}W_{2}^{T}<0, \end{array}$$

*then, if Z exists, the following holds:*

$$\begin{array}{c} W_{1}+W_{2}Z^{-1}W_{2}^{T}+W_{3}^{T}ZW_{3}<0. \end{array}$$

**Lemma** **2** **([23]).** *For given proper dimensioned matrices $M_{a}\left(a=1,2,3\right)$ and a scalar $\bar{\epsilon }>0$, if*

$$\begin{array}{c} M_{1}>0,\;M_{3}<0,{\bar{\epsilon }}^{2}M_{1}+\bar{\epsilon }M_{2}+M_{3}<0. \end{array}$$

*then, the inequality:*

$$\begin{array}{c} \epsilon^{2}M_{1}+\epsilon M_{2}+M_{3}<0, \end{array}$$

*holds for $\forall \epsilon \in (0,\bar{\epsilon }]$.*

**Definition** **1** **([24]).** *Given positive constants ϵ>0 and e∈(0,1). If it holds that:*

(10) $${E\{∥x\left(k\right)∥}^{2}\}\leq \epsilon e^{k}{E\{∥x\left(0\right)∥}^{2}\}$$

*then system (9) is called stochastically finite-time exponential stable with external disturbance.*

**Definition** **2** **([25]).** *Given a positive scalar μ>0, if system (9) satisfies the following conditions for all nonzero $w\left(k\right)\in l_{2}\left[0,\infty \right)$ when the initial conditions are zero:*

(11) $$\begin{array}{c} E\{\sum_{k=0}^{T}{∥y\left(k\right)∥}^{2}\}<\mu^{2}E\{\sum_{k=0}^{T}{∥\omega \left(k\right)∥}^{2}\} \end{array}$$

*where T is the upper bound of finite time, then the system is called a randomly stable system with w(k) disturbance decay.*

## 3. Main Results

### 3.1. The Stochastically Finite-Time Exponential Stable Analysis

**Theorem** **1.** *Suppose there are scalars μ>0, α∈[0,1], β∈[0,1] and $0<\tau_{m}\leq \tau_{M}$. If there exist symmetric positive definite matrices $R_{ap}>0$ and $U_{ap}>0$, such that $\forall a,b\in S_{1},m,n\in S_{2},p,q\in S_{3}$:*

(12) $$\begin{array}{c} \left[\begin{array}{cccc} U & 0 & H_{amp}^{T}{\bar{A}}_{amp} & H_{amp}^{T}B_{ap}\\ {}* & U & \bar{\alpha }H_{amp}^{T}E_{ap} & 0\\ {}* & * & -{\left(E_{\epsilon }U_{ap}E_{\epsilon }\right)}^{-1} & 0\\ {}* & * & * & E_{\epsilon }^{T}D_{a}E_{\epsilon }-\mu^{2}I \end{array}\right]<0, \end{array}$$

(13) $$\begin{array}{c} \beta \leq \lambda_{1}, \end{array}$$

(14) $$\begin{array}{c} E_{\epsilon }^{T}D_{a}E_{\epsilon }<\mu^{2}I, \end{array}$$

*where $H_{amp}=\left[\sqrt{\pi_{a1}\phi_{m1}\psi_{p1}^{1}}I,\ldots ,\sqrt{\pi_{ab}\phi_{mn}\psi_{pq}^{b}}I,\ldots ,\sqrt{\pi_{as_{1}}\phi_{ms_{2}}\psi_{ps_{3}}^{s_{1}}}I\right]$, $\bar{\alpha }=\sqrt{\alpha (1-\alpha )}$, $\lambda_{1}=\lambda_{\max}\left(P_{ap}\right)$, ${\bar{A}}_{amp}=A_{a}+\left(1-\alpha \right)C_{a}\left(K_{p}+\Delta K_{p}\right)\left(1+\Delta_{m}\right)+F_{a}^{T}F_{a}$, $B_{ap}=B_{a}+\left(K_{dp}+\Delta K_{dp}\right)$, $E_{ap}=-C_{a}\left(K_{p}+\Delta K_{p}\right)E_{\epsilon }\left(I+\Delta_{m}\right)$, $U=diag\{-U_{11},\ldots ,-U_{bq},\ldots ,U_{ap}\}$.*

*Then the MJSPSs (SMJSclosed) satisfies the H_∞_ performance stability.*

**Proof.**  The ensuing Lyapunov–Krasovskii functional is established as follows:

(15) $$\begin{array}{c} V\left(k\right)=x^{T}\left(k\right)P_{\sigma (k),\varphi (k)}x\left(k\right), \end{array}$$

where $P_{\sigma (k),\varphi (k)}>0$. (σ(k),φ(k)) takes values in (a,p), $\forall \in S_{1}\times S_{2}$ and obeys the DMC it follows that

(16) $$\begin{array}{cc} \; & \mathrm{Pr}\{\sigma (k+1)=b,\varphi (k+1)=q|\sigma (k)=a,\varphi (k)=p\}\\ \; & =\mathrm{Pr}\left\{\sigma \left(k+1\right)=b|\sigma \left(k\right)=a,\delta_{k}=p\right\}\times \mathrm{Pr}\left\{\varphi \left(k+1\right)=q|\sigma \left(k+1\right)=b,\sigma \left(k\right)=a,\psi \left(k\right)=p\right\}\\ \; & =\pi_{ab}\psi_{pq}^{b}. \end{array}$$

By evaluating the difference of V(k) in Equation (15), then the mathematical expectation manifests as

(17) $$\begin{array}{cc} E\{\Delta V(k)\}= & E\{V(k+1)-V(k)\}\\ = & E\{x^{T}\left(k+1\right)\sum_{b\in S_{1},q\in S_{2}}\mathrm{Pr}\left\{\sigma \left(k+1\right)=b,\varphi \left(k+1\right)=q|\sigma \left(k\right)=a,\varphi \left(k\right)=p\right\}\\ \; & P_{bq}x\left(k+1\right)-x^{T}\left(k\right)P_{ap}x\left(k\right)\}\\ = & E\{x^{T}\left(k+1\right)\sum_{b\in S_{1}}\pi_{ab}\sum_{q\in S_{2}}\psi_{pq}^{b}P_{bq}x\left(k+1\right)-x^{T}\left(k\right)P_{ap}x\left(k\right)\}\\ = & E\{x^{T}\left(k+1\right)P_{ap}x\left(k+1\right)-x^{T}\left(k\right)P_{ap}x\left(k\right)\} \end{array}$$

where $P_{ap}=\sum_{b\in S_{1}}\pi_{ab}\sum_{q\in S_{2}}\psi_{pq}^{b}P_{bq}$. Combining Equation (1), one can get

(18) $$\begin{array}{cc} E\{\Delta V(k)\}= & E\{\xi^{T}\left(k\right)\Upsilon_{1amp}^{T}E_{\epsilon }^{T}P_{ap}E_{\epsilon }\Upsilon_{1amp}\xi \left(k\right)+\xi^{T}\left(k\right)\Upsilon_{2amp}^{T}E_{\epsilon }^{T}P_{ap}E_{\epsilon }\Upsilon_{2amp}\xi \left(k\right)\\ + & w^{T}\left(k\right)E_{\epsilon }^{T}D_{a}E_{\epsilon }w^{T}\left(k\right)-\xi^{T}\left(k\right)P_{ap}\xi \left(k\right)\} \end{array}$$

where $\xi \left(k\right)=[x^{T}\left(k\right),\;x^{T}\left(k-\tau \left(k\right)\right)]$, $\Upsilon_{1amp}=\left[A_{a}+\left(1-\alpha \right)C_{a}\left(K_{p}+\Delta K_{p}\right)\left(1+\Delta_{m}\right),\;B_{a}+\left(K_{dp}+\Delta K_{dp}\right)\right]$, $\Upsilon_{2amp}=\sqrt{\alpha (1-\alpha )}\left[-C_{a}\left(K_{p}+\Delta K_{p}\right)\left(I+\Delta_{m}\right),\;0\right]$.

Setting $P_{ap}={\left(E_{\epsilon }U_{ap}E_{\epsilon }\right)}^{-1}$, one has

(19) $$\begin{array}{cc} E\{\Delta V(k)\}= & E\{\xi^{T}\left(k\right)\left[\Upsilon_{1amp}^{T}E_{\epsilon }^{T}P_{ap}E_{\epsilon }\Upsilon_{1amp}+\Upsilon_{2amp}^{T}E_{\epsilon }^{T}P_{ap}E_{\epsilon }\Upsilon_{2amp}+\Upsilon_{3ap}\right]\xi \left(k\right)+w\} \end{array}$$

where $w=\lambda_{\max}\left\{E_{\epsilon }^{T}D_{a}E_{\epsilon }\right\}\bar{w}$.

For any scalar β>0, it follows from Equation (12) that

(20) $$\begin{array}{c} E\left\{\Delta V\left(k\right)\right\}\leq -\beta E\{∥x\left(k\right){∥}^{2}\}+E\left\{w\right\}. \end{array}$$

According to Equation (15), we obtain

(21) $$\begin{array}{c} E\left\{\Delta V\left(k\right)\right\}\leq \lambda_{\max}\left(P_{ap}\right)E\{∥x\left(k\right){∥}^{2}\}=\lambda_{1}E\{∥x\left(k\right){∥}^{2}\}, \end{array}$$

(22) $$\begin{array}{c} E\left\{\Delta V\left(k\right)\right\}\geq \lambda_{\min}\left(P_{ap}\right)E\{∥x\left(k\right){∥}^{2}\}=\lambda_{2}E\{∥x\left(k\right){∥}^{2}\}. \end{array}$$

Combining Equations (20) and (21), it can be derived for any scalar χ>1 that the following equality holds:

(23) $$\begin{array}{cc} \; & \chi^{k+1}E\left\{V\left(x\left(k+1\right)\right)\right\}-\chi^{k}E\left\{V\left(x\left(k\right)\right)\right\}\\ = & \chi^{k+1}\left(E\left\{V\left(x\left(k+1\right)\right)\right\}-E\left\{V\left(x\left(k\right)\right)\right\}\right)+\chi^{k}\left(\chi -1\right)E\left\{V\left(x\left(k\right)\right)\right\}\\ \leq & -\beta \chi^{k+1}{E\{∥x\left(k\right)∥}^{2}\}+\chi^{k}E\left\{w\right\}+\chi^{k}{\left(\chi -1\right)\beta E\{∥x\left(k\right)∥}^{2}\}\\ = & \chi^{k}{h\left(\chi \right)E\{∥x\left(k\right)∥}^{2}\}+\chi^{k}E\left\{w\right\} \end{array}$$

where $h\left(\chi \right)=-\beta \chi +\lambda_{1}\left(\chi -1\right)$. By summing both sides of Equation (23) from 0 to T−1 and rearranging yields

(24) $$\begin{array}{c} \chi^{T}V\left(k\right)-V\left(0\right)\leq h\left(\chi \right)\sum_{k=0}^{T}\chi^{k}{∥e\left(k\right)∥}^{2}+\frac{\chi (\chi^{T}-1)}{\chi -1}w \end{array}$$

Due to $V\left(0\right)\leq -{\beta ∥x\left(0\right)∥}^{2}$. One can obtain

(25) $$\begin{array}{c} {∥x\left(k\right)∥}^{2}\leq \frac{h\left(\chi \right)\chi^{-T}}{\lambda_{2}}\sum_{k=0}^{T}\chi^{k}{∥x\left(k\right)∥}^{2}+\frac{\chi (\chi^{T}-1)}{\chi -1}w+\frac{\beta \chi^{-T}}{\lambda_{2}}{∥x\left(0\right)∥}^{2} \end{array}$$

Evidently, $\lim_{\chi \to \infty }h\left(\chi \right)\to 0$. The above equation can be further simplified to

(26) $$\begin{array}{c} {∥x\left(k\right)∥}^{2}\leq \frac{\beta \chi^{-T}}{\lambda_{2}}{∥x\left(0\right)∥}^{2}+\frac{\chi (\chi^{T}-1)}{\chi -1}w \end{array}$$

which satisfies Definition 1, thereby ensuring that the system (9) can achieve exponential convergence.

Next, we analyze the *H*_∞_ performance index of the system as follows:

(27) $$\begin{array}{c} J=E\{\Delta V\left(k\right)+y^{T}\left(k\right)y\left(k\right)-\mu^{2}w^{T}\left(k\right)w\left(k\right)\}. \end{array}$$

Using the Schur complement, one has

(28) $$\begin{array}{cc} J= & E\{\xi^{T}\left(k\right)\left[{\bar{\Upsilon }}_{1amp}^{T}E_{\epsilon }^{T}P_{ap}E_{\epsilon }{\bar{\Upsilon }}_{1amp}+\Upsilon_{2amp}^{T}E_{\epsilon }^{T}P_{ap}E_{\epsilon }\Upsilon_{2amp}+\Upsilon_{3ap}\right]\xi \left(k\right)\} \end{array}$$

where ${\bar{\Upsilon }}_{1amp}=\left[A_{a}+\left(1-\alpha \right)C_{a}\left(K_{p}+\Delta K_{p}\right)\left(1+\Delta_{m}\right)+F_{a}^{T}F_{a},\;B_{a}+\left(K_{dp}+\Delta K_{dp}\right)\right]$, $\Upsilon_{3ap}=diag\left\{-P_{ap},\;E_{\epsilon }^{T}D_{a}E_{\epsilon }-\mu^{2}I\right\}$.

Recalling (12), it can be obtained that

(29) $$\begin{array}{c} E\{\Delta V\left(k\right)+y^{T}\left(k\right)y\left(k\right)-\mu^{2}w^{T}\left(k\right)w\left(k\right)\}\leq 0. \end{array}$$

By summing both sides of Equation (29) from 0 to ∞, it has

(30) $$\begin{array}{c} E\left\{\sum_{k=0}^{\infty }y^{T}\left(k\right)y\left(k\right)-\mu^{2}\sum_{k=0}^{\infty }w^{T}\left(k\right)w\left(k\right)\right\}\leq E\left\{V\left(0\right)-V\left(\infty \right)\right\}. \end{array}$$

With zero initial conditions, we obtain

(31) $$\begin{array}{c} E\{\sum_{k=0}^{\infty }y^{T}\left(k\right)y\left(k\right)\leq \mu^{2}\sum_{k=0}^{\infty }w^{T}\left(k\right)w\left(k\right). \end{array}$$

Evidently, the *H*_∞_ performance condition (9) is met. □

### 3.2. Controller Design

**Theorem** **2.** *Suppose there are scalars μ>0, α∈[0,1], β∈[0,1] and $0<\tau_{m}\leq \tau_{M}$. If there exist symmetric positive definite matrices $\Omega_{ap}$, $U_{ap}>0$, and matrices $K_{p}$, $K_{dp}$, $U_{1p}$, $V_{1p}$, $U_{2p}$, $V_{2p}$, $Y_{ap}$, $Z_{ap}$ with proper dimensions, such that $\forall a,b\in S_{1},m,n\in S_{2},p,q\in S_{3}$, ι=1,2:*

(32) $$\begin{array}{c} \left[\begin{array}{ccc} \Sigma_{ap} & \Omega_{ap}^{1}R_{c} & \Omega_{ap}^{2T}\Xi_{e}\\ {}* & -R_{c} & 0\\ {}* & * & -R_{c} \end{array}\right]<0, \end{array}$$

*where*

$$\begin{array}{c} \Sigma_{ap}=\left[\begin{array}{cccc} U & 0 & H_{amp}^{T}{\tilde{A}}_{ap} & H_{amp}^{T}{\tilde{B}}_{ap}\\ {}* & U & \bar{\alpha }H_{amp}^{T}{\tilde{E}}_{ap} & 0\\ {}* & * & \Theta_{ap}^{\iota } & 0\\ {}* & * & * & E_{\epsilon }^{T}D_{a}E_{\epsilon }-\mu^{2}I \end{array}\right]<0, \end{array}$$

*$U=diag\{-U_{11},\ldots ,-U_{bq},\ldots ,U_{ap}\}$, $H_{amp}=\left[\sqrt{\pi_{a1}\phi_{m1}\psi_{p1}^{1}}I,\;\ldots ,\;\sqrt{\pi_{ab}\phi_{mn}\psi_{pq}^{b}}I,\;\ldots ,\;\sqrt{\pi_{as_{1}}\phi_{ms_{2}}\psi_{ps_{3}}^{s_{1}}}I\right]$, ${\tilde{A}}_{ap}=A_{a}Y_{ap}+\left(1-\alpha \right)C_{a}\left(K_{p}+U_{1p}V_{1p}\right)+F_{a}^{T}F_{a}$, ${\tilde{B}}_{ap}=B_{a}Z_{ap}+\left(K_{dp}+U_{2p}V_{2p}\right)$, ${\tilde{E}}_{ap}=-C_{a}\left(K_{p}+U_{1p}V_{1p}\right)$, $\bar{\alpha }=\sqrt{\alpha (1-\alpha )}$, $\lambda_{1}=\lambda_{\max}\left(P_{ap}\right)$, $\Omega_{amp}^{1}=\left[\left(1-\alpha \right)\sqrt{\pi_{a1}\phi_{m1}\psi_{p1}^{1}}B_{a}^{T},\;\ldots ,\;\left(1-\alpha \right)\sqrt{\pi_{as_{1}}\phi_{ms_{2}}\psi_{ps_{3}}^{s_{1}}}B_{a}^{T},\;\bar{\alpha }\sqrt{\pi_{a1}\phi_{m1}\psi_{p1}^{1}}B_{a}^{T},\ldots ,\bar{\alpha }\sqrt{\pi_{as_{1}}\phi_{ms_{2}}\psi_{ps_{3}}^{s_{1}}}B_{a}^{T},\;0,\;0\right]$, $\Omega_{amp}^{2}=\left[0,\ldots ,0,0,\ldots ,0,K_{p},\;0\right]$, $\Theta_{ap}^{1}=E_{\epsilon }U_{ap}E_{\epsilon }-Y_{ap}^{T}-Y_{ap}$, $\Theta_{ap}^{2}=I_{1}U_{ap}I_{1}-Y_{ap}^{T}-Y_{ap}$.*

*Then the MJSPSs (9) satisfies the H_∞_ performance stability. The controller gain is given by the following equation:*

(33) $$\begin{array}{cc} K_{p} & =K_{p}Y_{ap}^{-1},\\ K_{dp} & =K_{dp}Z_{ap}^{-1}. \end{array}$$

**Proof.**  Applying the Schur complement, Equation (32) can be simplified to

(34) $$\begin{array}{c} \Sigma_{ap}+{ℵ}_{ap}^{1}R_{q}{ℵ}_{ap}^{1T}+{ℵ}_{ap}^{2T}\Lambda R_{q}^{-1}\Lambda {ℵ}_{ap}^{2}<0, \end{array}$$

it further follows that

(35) $$\begin{array}{c} \Sigma_{ap}+{ℵ}_{ap}^{1}R_{q}{ℵ}_{ap}^{1T}+{ℵ}_{ap}^{2T}\Delta R_{q}^{-1}\Delta {ℵ}_{ap}^{2}<0 \end{array}$$

From Lemma 1, we have

(36) $$\begin{array}{c} \Sigma_{ap}+{ℵ}_{ap}^{1}\Delta {ℵ}_{ap}^{2}+{ℵ}_{ap}^{2T}\Delta {ℵ}_{ap}^{1T}<0 \end{array}$$

Equation (36) with ι=1 can be equivalently rewritten as

(37) $$\begin{array}{c} {\bar{\kappa }}^{2}\left[\begin{array}{cc} 0 & 0\\ {}* & \Xi_{ap}^{1} \end{array}\right]+\bar{\kappa }\left[\begin{array}{cc} 0 & 0\\ {}* & \Xi_{ap}^{2} \end{array}\right]+\left[\begin{array}{cc} \Upsilon_{ap}^{11} & \Upsilon_{ap}^{12}\\ {}* & \Xi_{ap}^{3} \end{array}\right]<0 \end{array}$$

where

$$\begin{array}{cc} \Upsilon_{ap}^{11} & =\mathrm{diag}\left\{U,\;U\right\},\Upsilon_{ap}^{12}=\left[\begin{array}{ccc} H_{amp}^{T}A_{amp}Y_{ap} & H_{amp}^{T}B_{ap}Z_{ap} & H_{amp}^{T}D_{a}Y_{ap}\\ \bar{\alpha }H_{amp}^{T}E_{ap}Y_{ap} & 0 & 0 \end{array}\right],I_{1}=\left[\begin{array}{cc} I_{n1} & 0\\ {}* & 0 \end{array}\right],\\ I_{2} & =\left[\begin{array}{cc} 0 & 0\\ {}* & I_{n2} \end{array}\right],\Xi_{ap}^{1}=\left[\begin{array}{cc} I_{2}U_{ap}I_{2} & 0\\ {}* & 0 \end{array}\right]\Xi_{ap}^{2}=\left[\begin{array}{cc} I_{1}U_{ap}I_{2}+I_{2}U_{ap}I_{1} & 0\\ {}* & 0 \end{array}\right],\\ \Xi_{ap}^{3} & =\left[\begin{array}{cc} I_{1}U_{ap}I_{1}-Y_{ap}^{T}-Y_{ap} & 0\\ {}* & I_{1}^{T}D_{a}I_{1}-\mu^{2}I \end{array}\right]. \end{array}$$

Due to $U_{ap}>0$, one obtains

(38) $$\begin{array}{c} \left[\begin{array}{cc} 0 & 0\\ {}* & \Xi_{ap}^{1} \end{array}\right]>0. \end{array}$$

Similarly, for Equation (36) with ι=2, it can be derived that

(39) $$\begin{array}{c} \left[\begin{array}{cc} \Upsilon_{ap}^{11} & \Upsilon_{ap}^{12}\\ {}* & \Xi_{ap}^{3} \end{array}\right]<0. \end{array}$$

From Lemma 2, it follows that

(40) $$\begin{array}{c} \kappa^{2}\left[\begin{array}{cc} 0 & 0\\ {}* & \Xi_{ap}^{1} \end{array}\right]+\kappa \left[\begin{array}{cc} 0 & 0\\ {}* & \Xi_{ap}^{2} \end{array}\right]+\left[\begin{array}{cc} \Upsilon_{ap}^{11} & \Upsilon_{ap}^{12}\\ {}* & \Xi_{ap}^{3} \end{array}\right]<0, \end{array}$$

that is

(41) $$\begin{array}{c} \left[\begin{array}{cc} \Upsilon_{ap}^{11} & \Upsilon_{ap}^{12}\\ {}* & \Upsilon_{ap}^{22} \end{array}\right]<0, \end{array}$$

where $\Upsilon_{ap}^{22}=diag\left\{E_{\epsilon }U_{ap}E_{\epsilon }-Y_{ap}^{T}-Y_{ap},\;E_{\epsilon }^{T}D_{a}E_{\epsilon }-\mu^{2}I\right\}$. Applying inequality $E_{\epsilon }U_{ap}E_{\epsilon }-Y_{ap}^{T}-Y_{ap}\leq -Y_{ap}^{T}\left(E_{\epsilon }U_{ap}E_{\epsilon }\right)Y_{ap}$, it implies that

(42) $$\begin{array}{c} \left[\begin{array}{cc} \Upsilon_{ap}^{11} & \Upsilon_{ap}^{12}\\ {}* & {\bar{\Upsilon }}_{ap}^{22} \end{array}\right]<0, \end{array}$$

where ${\bar{\Upsilon }}_{ap}^{22}=diag\left\{-Y_{ap}^{T}\left(E_{\epsilon }U_{ap}E_{\epsilon }\right)Y_{ap},\;E_{\epsilon }^{T}D_{a}E_{\epsilon }-\mu^{2}I\right\}$.

Pre- and postmultiplying (41) by $diag\{I,\ldots ,I,Y_{ap}^{-T},Z_{ap}^{-T},I\}$ and its transposition. Clearly, by (12), (42) holds. □

## 4. Examples

Consider an inverted pendulum system controlled by a DC motor through gear transmission [14,26], whose dynamic model can be described as

$$\left\{\begin{array}{c} {\dot{x}}_{1}\left(t\right)=x_{2}\left(t\right)\\ {\dot{x}}_{2}\left(t\right)=\frac{g}{l}\sin\left(x_{1}\left(t\right)\right)+\frac{NK_{m}}{ml^{2}}x_{3}\left(t\right)\\ L_{p}{\dot{x}}_{3}\left(t\right)=-K_{p}N_{p}x_{2}\left(t\right)-R_{p}x_{3}\left(t\right)+u\left(t\right)+w\left(t\right) \end{array}\right.$$

where $x_{2}\left(t\right)={\dot{\theta }}_{p}\left(t\right)$ is the angular velocity of the pendulum arm in the vertical direction. *g* is the gravitational constant, *l* represents the axis length, $x_{2}\left(t\right)={\dot{\theta }}_{p}\left(t\right)$ is the angle between the pendulum arm and the vertical direction, the gear ratio is expressed as $N_{r}$, the motor torque constant is $K_{m}$, and $x_{3}\left(t\right)=I_{a}\left(t\right)$ is the motor current. Other system parameters include inductance $L_{a}$, motor torque constant $K_{b}$, resistance $R_{i}$, control input voltage u(t), and external disturbance w(t). The specific values of the parameters are as follows: $g=9.8\;{m/s}^{2},K_{m}=0.1\;N\times \;m/s,K_{b}=0.1\;V\times \;s/\mathrm{rad},N=10,l=1\;m,L_{a}=50\;\mathrm{mH}$. By setting the singular perturbation parameter $\epsilon =L_{a}$ inductance, the linear model of the system can be obtained:

$$E_{\epsilon }\dot{x}\left(t\right)=A_{\sigma (t)}x\left(t\right)+B_{\sigma (t)}E_{\epsilon }\left(x\left(t\right)-\tau \left(t\right)\right)+C_{\sigma (t)}u\left(t\right)+D_{\sigma (t)}w\left(t\right),$$

where $A_{1}=\left[\begin{array}{ccc} 0 & 1 & 0\\ \frac{g}{l} & 0 & \frac{NK_{m}}{ml^{2}}\\ 0 & -K_{b}N_{r} & -R_{1} \end{array}\right]$, $A_{2}=\left[\begin{array}{ccc} 0 & 1 & 0\\ \frac{g}{l} & 0 & \frac{NK_{m}}{ml^{2}}\\ 0 & -K_{b}N_{r} & -R_{2} \end{array}\right]$, $B_{1}=D_{1}=\left[\begin{array}{c} 0\\ 1\\ 1 \end{array}\right]$, $B_{2}=D_{2}=\left[\begin{array}{c} 0\\ 0.5\\ 0.5 \end{array}\right]$, $C_{1}=0.5I$, $C_{2}=0.1I$, $E_{\epsilon }=\mathrm{diag}\left\{1,1,\epsilon \right\}$.

According to the Euler discrete method, by discretizing the continuous system with a sampling period of T=0.08s, we can obtain the parameters of the singular Markov jump discrete complex network as follows:

$$E_{\epsilon }x\left(k+1\right)=A_{a}x\left(k\right)+B_{a}E_{\epsilon }\left(x\left(k\right)-\tau \left(k\right)\right)+C_{a}u\left(k\right)+D_{a}w\left(k\right),$$

where $A_{1}=\left[\begin{array}{ccc} 1.0313 & 0.0796 & 0.0020\\ 0.7805 & 0.9913 & 0.0397\\ -0.0196 & -0.0397 & 0.0089 \end{array}\right]$, $A_{2}=\left[\begin{array}{ccc} 1.0313 & 0.0797 & 0.0018\\ 0.7812 & 0.9944 & 0.0354\\ -0.0181 & -0.0354 & 0.0063 \end{array}\right]$, $B_{1}=D_{1}=\left[\begin{array}{c} 0.0239\\ 0.7993\\ 0.7818 \end{array}\right]$, $B_{2}=D_{2}=\left[\begin{array}{c} 0.0180\\ 0.5596\\ 0.4708 \end{array}\right]$, $C_{1}=0.5I$, $C_{2}=0.1I$, $E_{\epsilon }=\mathrm{diag}\left\{1,1,0.05\right\}$. 

The Markov jump modes of the system, quantizer, and controller are shown in Figure 1, and the transition probabilities are controlled by the following transition probability matrix: $\Gamma_{1}=\left[\begin{array}{cc} 0.30 & 0.70\\ 0.50 & 0.50 \end{array}\right]$, $\Gamma_{2}^{1}=\left[\begin{array}{cc} 0.40 & 0.60\\ 0.60 & 0.40 \end{array}\right]$, $\Gamma_{2}^{2}=\left[\begin{array}{cc} 0.70 & 0.30\\ 0.65 & 0.35 \end{array}\right]$, $\Gamma_{3}=\left[\begin{array}{cc} 0.25 & 0.75\\ 0.55 & 0.45 \end{array}\right]$.

In the simulation, the external disturbance w(k) is chosen as an exponentially decaying signal: 0.05exp(−1.16k)sin(0.9πk). time delay τ(k) is [sin(k∗π/2)+1], The quantizer parameters $\rho_{1}$ and $\rho_{2}$ are 0.9 and 0.75, respectively. Other parameters are set as follows: $O_{1}\left(k\right)=0.05\cos\left(k\right)$, $O_{2}\left(k\right)=\sin\left(k\right)$, $U_{11}=\left[0.02,\;-0.01,\;0.01\right]$, $U_{12}=\left[0.03,\;0.04,\;0.02\right]$, $U_{21}=\left[0.10,\;0.10,\;0.10\right]$, $U_{22}=\left[0.20,\;0.20,\;0.20\right]$, $V_{11}=\left[\begin{array}{ccc} 0.03 & 0 & 0\\ 0 & -0.03 & 0\\ 0 & 0 & 0.026 \end{array}\right]$, $V_{12}=\left[\begin{array}{ccc} 0.04 & 0 & 0\\ 0 & -0.03 & 0\\ 0 & 0 & 0.018 \end{array}\right]$, $V_{21}=\left[\begin{array}{ccc} 0.1 & 0 & 0\\ 0 & -0.1 & 0\\ 0 & 0 & 0.05 \end{array}\right]$, and $V_{22}=\left[\begin{array}{ccc} 0.2 & 0 & 0\\ 0 & -0.2 & 0\\ 0 & 0 & 0.15 \end{array}\right]$. 

By solving the linear matrix inequality in Theorem 2, we can obtain the controller gain as

$$\begin{array}{cc} K_{1}=\left[-15.2679-1.9610-0.0100\right],\;\; & K_{2}=\left[-10.0853-1.5000-0.1500\right],\\ K_{d1}=\left[-19.04541.044510.8901\right],\;\; & K_{d2}=\left[-0.12520.14578.8815\right], \end{array}$$

**Figure 2:**
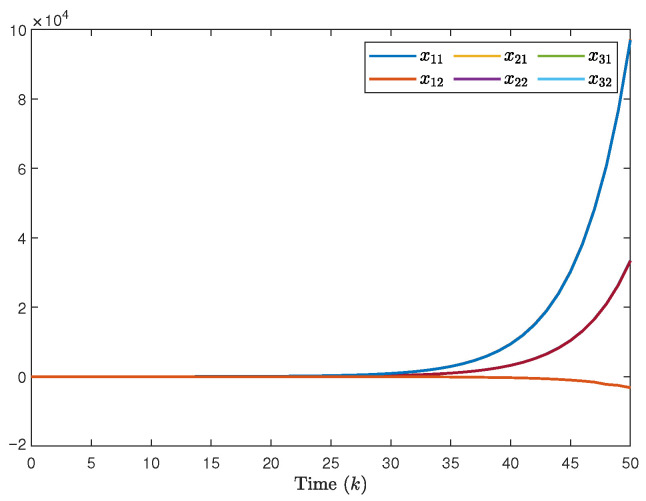
The state trajectory of the MJSPSs without controller.

**Figure 3:**
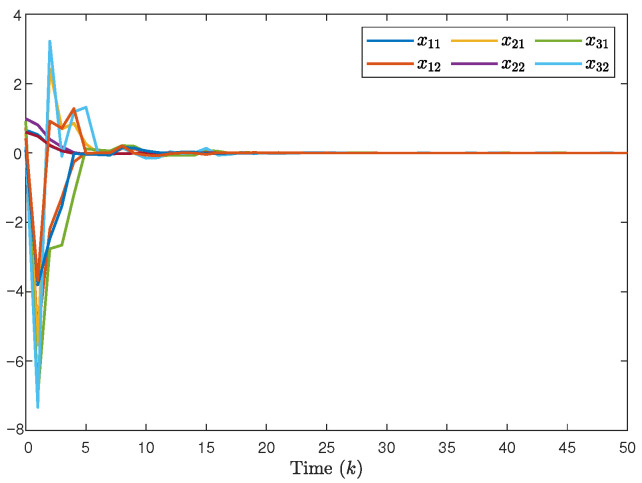
The state trajectory of the closed-loop MJSPSs.

**Figure 4:**
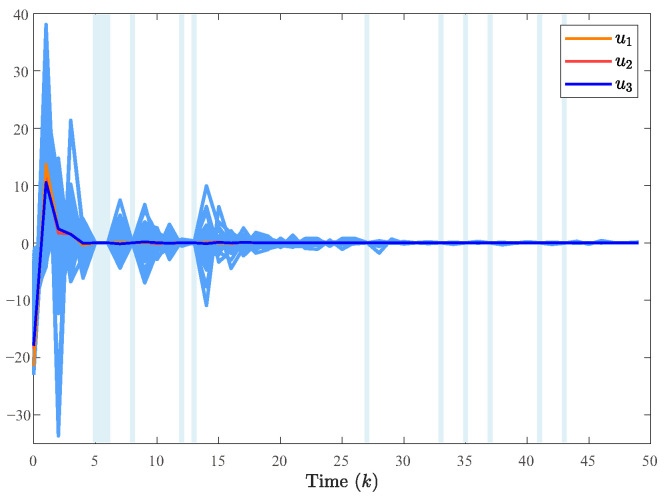
The control output curves and its average value in 100 simulation. The dark blue color represents the results of 100 simulations. The light blue color represents the DOS attack sequence.

**Figure 5:**
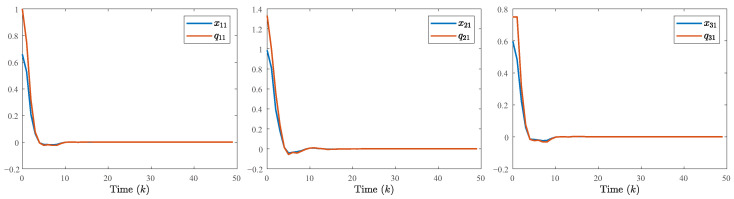
Comparison of the state input and quantization input curves.

**Figure 6:**
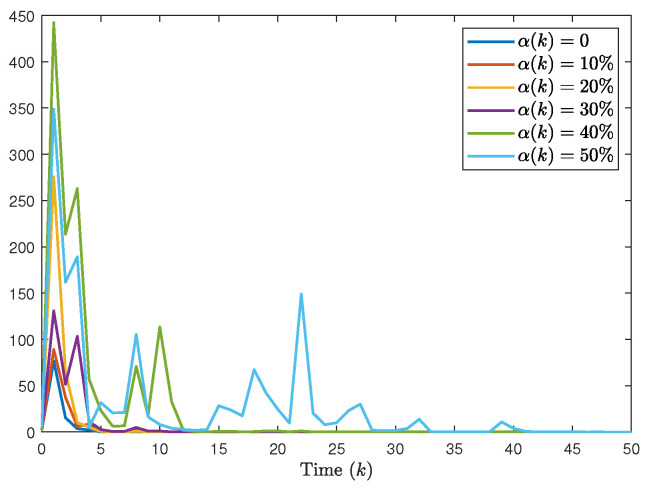
The state $x^{T}\left(k\right)x\left(k\right)$ curve under different attack frequencies.

**Figure 7:**
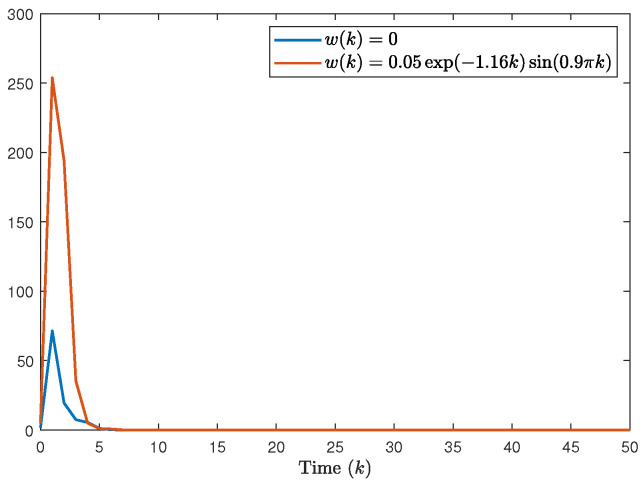
The state $x^{T}\left(k\right)x\left(k\right)$ curve with and without disturbance.

The system generates random sequences to produce target node values within the range [0, 1]. As illustrated in Figure 2, in the absence of a controller, the state trajectories of individual nodes exhibit divergent behavior and cannot be effectively tracked. An in-depth analysis of experimental data from the closed-loop system, presented in Figure 3, Figure 4 and Figure 5, demonstrates the performance under complex operating conditions, including external disturbances and actuator failures. Figure 4 shows the simulation of 100 of the control output curves and DoS attack sequences. The results indicate that the four network nodes successfully achieve rapid tracking of the target node’s state trajectory by employing the designed non-fragile controller parameters. Furthermore, as shown in Figure 3, Figure 4 and Figure 5, the state error between the four network nodes and the target node converges to a bounded region within a finite time, T=20 s. Figure 6 shows the state $x^{T}\left(k\right)x\left(k\right)$ curve under different attack frequencies α(k). These experimental results confirm the effectiveness and robustness of the proposed control method. As illustrated in Figure 7, the state trajectories $x^{T}\left(k\right)x\left(k\right)$ are compared with and without external disturbance. It is evident that the disturbance injects additional energy into the system, resulting in higher peaks during the transient phase. However, under the proposed non-fragile $H_{\infty }$ control, the system effectively suppresses this spectral energy, and the state trajectories rapidly converge to zero, satisfying the robust performance requirements.

**Figure 8:**
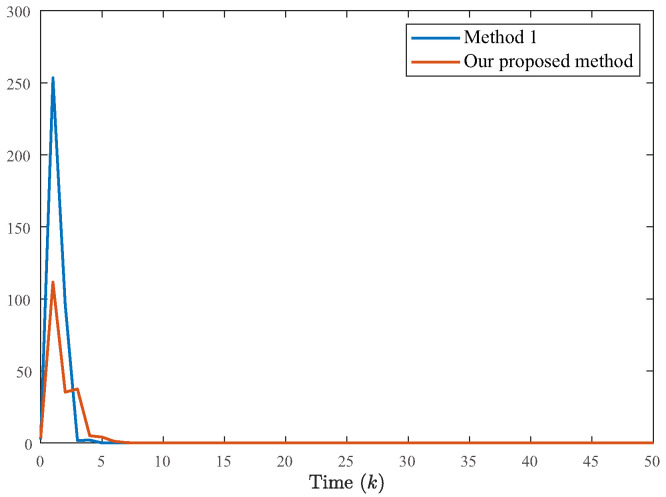
The state $x^{T}\left(k\right)x\left(k\right)$ curve under quantization density. Method 1 represents the fixed quantization density method [7,17,22], where ρ = 0.75. The quantitative densities of the method proposed in this article are $\rho_{1}$ = 0.95 and $\rho_{2}$ = 0.75.

To further highlight the advantages of the proposed strategy, this study employs a comparative experiment using the classical fixed quantization density method [7,17,22]. As shown in Figure 8, the proposed method with variable quantization density demonstrates faster convergence and superior interference suppression capabilities. This confirms that the ability to dynamically adjust quantization density enables the controller to maintain high performance even under Markov mode modeling, whereas the fixed strategy struggles to adapt.

## 5. Conclusions

This study addresses the asynchronous non-fragile $H_{\infty }$ control problem for time-delayed MJSPSs with variable quantization density and DoS attacks. By modeling the asynchronous dynamics between the system and controller modes using independent Markov chains, a robust control framework is proposed to ensure stochastic finite-time exponential stability and $H_{\infty }$ performance under conditions of delay, singular disturbances, and quantization uncertainty. Sufficient conditions are derived based on mode-dependent Lyapunov–Krasovskii functions, enabling the design of non-fragile controller gains to enhance system robustness. The effectiveness of this method is validated in bandwidth-constrained network environments. Future research will explore extending this framework to hidden-Markov jump singular disturbance systems and combining it with adaptive quantization strategies to further optimize performance under complex communication constraints.

## Data Availability

The data that support the findings of this study are available within the article.
